# Redefining mechanical ventilation in thoracic anesthesia: flow–controlled ventilation from bench to bedside—a narrative review

**DOI:** 10.3389/fmed.2026.1950213

**Published:** 2026-09-25

**Authors:** Timur Yurttas, Andrea Stieger, Martin Scharffenberg, Jakob Wittenstein

**Affiliations:** 1 Department of Anesthesiology, Rescue– and Pain Medicine, Cantonal Hospital St. Gallen, HOCH Health Ostschweiz, St. Gallen, Switzerland; 2 Department of Anesthesiology, Amsterdam University Medical Center, Amsterdam, Netherlands; 3 Department of Anesthesiology and Intensive Care Medicine, Pulmonary Engineering Group, Faculty of Medicine and University Hospital Carl Gustav Carus, TUD Dresden University of Technology, Dresden, Germany

**Keywords:** flow–controlled ventilation, mechanical power, one–lung ventilation, postoperative pulmonary complications, thoracic anesthesia, ventilator–induced lung injury

## Abstract

One–lung ventilation (OLV) remains a cornerstone of thoracic anesthesia but continues to challenge perioperative respiratory management. Restriction of ventilation to a reduced functional lung volume promotes ventilation–perfusion mismatch, alveolar instability, and exposure of the dependent lung to increased mechanical stress. Although contemporary lung–protective ventilation strategies have evolved considerably, postoperative pulmonary complications remain common, highlighting the need for innovative approaches to respiratory support. Flow–controlled ventilation (FCV) introduces a distinct physiological concept by actively controlling both inspiration and expiration through constant gas flow. Unlike conventional ventilation modes, which rely on passive exhalation, FCV enables linear pressure changes throughout the respiratory cycle and combines active expiratory control with direct intratracheal pressure monitoring and compliance–guided ventilator titration. This approach aims to improve ventilation efficiency while reducing mechanisms implicated in ventilator–induced lung injury. In this narrative review, we examine the physiological principles underlying FCV, discuss the technological innovations enabling its clinical application, and summarize current evidence from bench, preclinical, and clinical investigations. Experimental studies demonstrate improvements in respiratory mechanics, lung aeration, oxygenation, and mechanical power compared with conventional ventilation. Limited clinical data during OLV suggest potential improvements in oxygenation and carbon dioxide elimination at lower minute ventilation requirements; however, these findings are derived from small studies primarily evaluating physiological endpoints. Despite encouraging physiological results, important evidence gaps remain. Current clinical studies are limited by small sample sizes and focus predominantly on surrogate physiological outcomes. It remains unknown whether the observed improvements will translate into reductions in postoperative pulmonary complications or other patient–centered outcomes. By shifting attention from inspiration alone to the entire respiratory cycle, FCV challenges conventional paradigms of perioperative ventilation. Future multicenter outcome–driven trials will determine whether active expiratory control represents a meaningful advance in thoracic anesthesia or primarily a physiological innovation with limited clinical impact.

## Introduction

One–lung ventilation (OLV) remains one of the most challenging forms of intraoperative respiratory support. During thoracic surgery, ventilation must be maintained through a substantially reduced functional lung volume while simultaneously accommodating profound alterations in pulmonary physiology, including intrapulmonary shunt formation, ventilation–perfusion mismatch, altered respiratory mechanics, and increased susceptibility to hypoxemia (1). Although advances in anesthetic and surgical techniques have improved perioperative outcomes, postoperative pulmonary complications (PPCs) continue to represent a major source of morbidity following thoracic surgery and remain closely linked to prolonged hospitalization, increased healthcare utilization, and mortality (2).

Over the past two decades, increasing attention has focused on the contribution of intraoperative mechanical ventilation to pulmonary injury. Traditionally, lung–protective ventilation during OLV has centered on limiting tidal volume and airway pressures. More recently, the understanding of ventilator–induced lung injury (VILI) has evolved considerably, shifting from isolated ventilatory variables toward integrative physiological concepts such as driving pressure, mechanical power, cumulative energy exposure, and oxygen–associated toxicity. Contemporary ventilation strategies therefore increasingly aim to minimize both mechanical and biochemical stress while maintaining adequate gas exchange (3).

Despite these developments, important limitations remain. Even when contemporary protective ventilation strategies are applied, OLV continues to expose the ventilated lung to substantial stress and strain because the entire ventilatory load is delivered to a markedly reduced lung volume. Furthermore, most conventional ventilation modes actively control inspiration while leaving expiration entirely passive. Consequently, rapid expiratory flow and abrupt pressure decay remain inherent characteristics of both volume–controlled ventilation (VCV) and pressure–controlled ventilation (PCV). Emerging evidence suggests that these expiratory dynamics may contribute to cyclic alveolar collapse, energy dissipation, and regional mechanical stress, particularly in mechanically vulnerable lungs (4).

Flow–controlled ventilation (FCV) represents a distinct approach to mechanical ventilation by actively controlling both inspiration and expiration (5) (Figure 1). Combined with intratracheal pressure monitoring and individualized compliance–guided ventilator titration, FCV seeks to optimize gas exchange while reducing mechanical stress imposed on the respiratory system (6).

**Figure 1:**
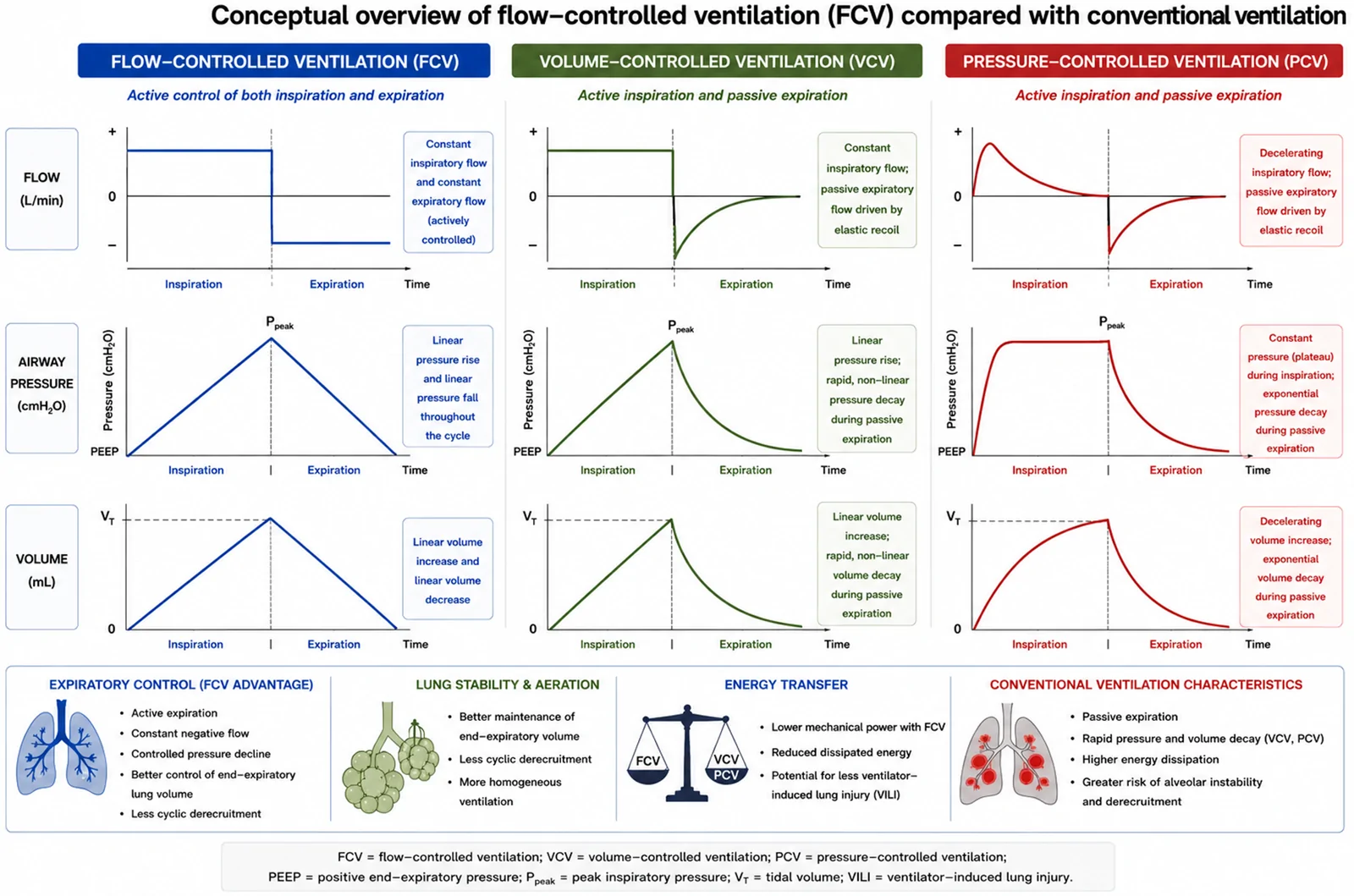
Conceptual overview of flow–controlled ventilation (FCV) compared with conventional ventilation. FCV, flow–controlled ventilation; VCV, volume–controlled ventilation; PCV, pressure–controlled ventilation; PEEP, positive end–expiratory pressure; P_peak_, peak inspiratory pressure; V_T_, tidal volume; VILI, ventilator–induced lung injury.

Since its introduction, FCV has been evaluated across a growing spectrum of experimental, preclinical, and clinical investigations. Studies in animal models have demonstrated improvements in oxygenation, respiratory mechanics, lung aeration, and mechanical energy transfer compared with conventional ventilation approaches (7–10). More recently, clinical investigations during thoracic surgery and OLV have reported encouraging physiological findings, including improved oxygenation, enhanced carbon dioxide elimination, lower minute ventilation requirements, and reductions in mechanical power (11, 12). Nevertheless, the available evidence remains limited, and the effects of FCV on PPCs and other patient–centered outcomes remain uncertain.

To identify relevant literature for this narrative review, PubMed/MEDLINE and Embase were searched using combinations of the terms “flow–controlled ventilation”, “flow controlled ventilation”, “FCV”, “thoracic anesthesia”, and “one–lung ventilation”. Publications considered relevant to the physiological principles, technical aspects, preclinical evidence, and clinical application of FCV, with particular emphasis on thoracic anesthesia and OLV, were included. Additional relevant publications were identified from the reference lists of retrieved articles. Given the narrative nature of this review, study selection was based on relevance to the scope of the review rather than predefined systematic inclusion and exclusion criteria.

## Clinical background and unmet needs in thoracic anesthesia

### OLV: current standards and physiological challenges

OLV is an essential component of modern thoracic anesthesia, enabling surgical exposure for lung resection, esophageal surgery, and increasingly complex minimally invasive procedures. However, OLV profoundly alters respiratory physiology. Following lung isolation, ventilation is restricted to a substantially reduced functional lung volume, while perfusion persists in both lungs. As a result, ventilation–perfusion mismatch and intrapulmonary shunt formation increase, often leading to impaired oxygenation and necessitating elevated inspired oxygen fractions (13).

Beyond gas exchange abnormalities, OLV substantially alters respiratory mechanics. The ventilated dependent lung receives the entirety of minute ventilation despite representing only a fraction of the original functional lung volume. Simultaneously, lateral positioning, surgical manipulation, mediastinal displacement, and diaphragmatic dysfunction further reduce lung compliance and promote regional heterogeneity of ventilation. Consequently, mechanical ventilation during OLV exposes the remaining ventilated lung to increased stress and strain, potentially amplifying the risk of VILI (3, 13, 14).

Despite advances in perioperative care, PPCs remain among the most frequent adverse events following thoracic surgery. Depending on patient characteristics, surgical complexity, and outcome definitions, PPCs occur in up to 20%–50% of patients and are associated with prolonged hospitalization, increased healthcare costs, and mortality (15, 16). These observations have driven extensive efforts to optimize intraoperative ventilation during OLV and minimize VILI (16).

### Mechanisms of VILI during OLV

VILI is increasingly recognized as a significant link in the development of PPCs following intraoperative ventilation. Traditionally, VILI has been attributed to several overlapping mechanisms, including volutrauma, barotrauma, atelectrauma, and biotrauma.

Volutrauma occurs when excessive tidal deformation results in overdistension of alveolar structures. Barotrauma refers to injury associated with elevated transpulmonary pressures, whereas atelectrauma arises from repetitive opening and closing of unstable lung units. These mechanical insults trigger inflammatory signaling pathways, resulting in local and systemic release of cytokines and inflammatory mediators, a process commonly referred to as biotrauma (2, 13, 17).

Importantly, OLV may amplify each of these injury mechanisms. Because ventilation is delivered to a substantially reduced lung volume, even seemingly protective ventilator settings may generate considerable regional stress and strain. Experimental and clinical studies suggest that the “baby lung” concept originally described in acute respiratory distress syndrome may also apply during OLV, where the functional lung available for ventilation is markedly reduced (18, 19). Consequently, global airway pressures and tidal volumes may underestimate the true mechanical burden experienced by individual lung regions.

More recently, the understanding of VILI has expanded beyond isolated pressure and volume variables. Driving pressure has emerged as an important surrogate marker of lung stress and has been associated with PPCs across a wide range of surgical populations (20). Similarly, mechanical power has been proposed as a comprehensive measure integrating tidal volume, airway pressures, respiratory rate, flow, and positive end–expiratory pressure (PEEP) into a single estimate of energy transferred to the respiratory system over time. During OLV, where ventilation is concentrated within a smaller functional lung volume, cumulative energy exposure may be particularly relevant (11, 21).

Complementing the concept of mechanical power, chemical power has recently been proposed to capture the biochemical burden of mechanical ventilation, including hyperoxia, oxidative stress, and carbon dioxide–related acid–base disturbances (4, 22). This concept is particularly relevant during OLV, where high inspired oxygen fractions are often required to maintain adequate oxygenation.

### Contemporary principles of lung–protective ventilation during OLV

Current recommendations for protective OLV emphasize the use of lower tidal volumes, limitation of driving pressure, avoidance of excessive inspired oxygen fractions, and application of moderate PEEP. These principles have largely replaced historical approaches that relied on tidal volumes exceeding 8–10 mL·kg^−1^ predicted body weight (16).

Among these strategies, lower tidal volume ventilation has become widely accepted as a cornerstone of protective OLV. Meta–analyses have demonstrated improvements in oxygenation and reductions in airway pressures with lower tidal volume approaches, although consistent reductions in PPCs remain less certain (23). Similarly, increasing attention has focused on individualized PEEP strategies aimed at balancing alveolar recruitment against overdistension. Recent randomized trials and meta–analyses suggest that individualized PEEP may improve intraoperative oxygenation and respiratory mechanics; however, effects on PPCs remain inconsistent (16, 24).

The PROTHOR trial, the largest randomized trial conducted in thoracic surgery to date, demonstrated that higher PEEP combined with recruitment maneuvers improved respiratory mechanics and oxygenation but failed to reduce PPCs when compared with lower PEEP. Furthermore, higher PEEP was associated with increased hemodynamic instability and vasoactive support requirements. Together, these findings suggest that improvements in intraoperative physiological variables do not necessarily translate into improved postoperative outcomes (16).

### Persistent unmet needs

Despite substantial progress in protective ventilation, important challenges remain unresolved. Hypoxemia, cyclic alveolar collapse, elevated mechanical power, and PPC continue to occur even when contemporary protective ventilation strategies are applied. Furthermore, many interventions that improve oxygenation and respiratory mechanics fail to consistently reduce PPCs (4).

One potential limitation of conventional ventilation is its predominant focus on inspiration, while expiration remains passive (5).

Emerging evidence suggests that these expiratory dynamics may contribute substantially to regional derecruitment, energy dissipation, and mechanical stress within vulnerable lung units. Consequently, active control of expiration has gained increasing interest as a potential strategy to improve ventilation homogeneity and reduce VILI. These observations have stimulated interest in active expiratory control as a potential component of lung–protective ventilation (25).

### Why expiration matters: the physiological foundation of FCV

Although modern ventilation strategies have evolved substantially over recent decades, the fundamental characteristics of expiration have remained largely unchanged. During conventional VCV and PCV, expiration occurs passively after termination of inspiratory flow. The pressure gradient between the alveoli and airway opening drives gas out of the lungs, resulting in an initially high expiratory flow that progressively declines as airway pressure approaches PEEP. This process is characterized by an exponential decrease in both flow and airway pressure, with the majority of expiratory volume being expelled during the early phase of expiration (26).

From a physiological perspective, passive expiration may have important consequences for lung stability. The rapid decline in airway pressure occurring immediately after inspiration may facilitate collapse of mechanically vulnerable, unstable lung units, particularly in dependent regions characterized by reduced compliance, increased superimposed pressure, or heterogeneous time constants. This phenomenon may be especially relevant during OLV, where the ventilated lung already operates close to the limits of recruitability because of reduced functional lung volume, surgical manipulation, and altered chest wall mechanics (27).

Experimental studies have demonstrated that alveolar recruitment and derecruitment are dynamic processes occurring throughout the respiratory cycle rather than static phenomena determined solely by end–inspiratory or end–expiratory pressures. Consequently, the trajectory of pressure decline during expiration may influence the stability of alveolar units and the extent of cyclic recruitment–derecruitment. Repetitive opening and closing of unstable alveoli is considered a major contributor to atelectrauma, one of the central mechanisms of VILI (28, 29).

In addition to promoting alveolar instability, passive expiration may contribute to energy dissipation within the respiratory system. During mechanical ventilation, not all delivered energy is recovered during expiration. A proportion of the applied energy is dissipated within lung tissue through frictional forces, tissue deformation, and repetitive recruitment–derecruitment phenomena. Experimental evidence suggests that this dissipated energy may represent an important determinant of VILI and may provide a mechanistic link between respiratory mechanics and biological injury (30).

The concept of mechanical power has further expanded this understanding. Increasing evidence suggests that mechanical power may predict pulmonary complications more accurately than individual ventilatory variables alone. However, conventional mechanical power calculations primarily focus on inspiratory energy delivery and provide only limited insight into the role of expiratory mechanics and energy dissipation occurring during expiration (3, 31).

These observations raise an important question: can expiration itself become a therapeutic target?

FCV was developed to address precisely this question. In contrast to conventional ventilation, FCV maintains continuous gas flow throughout the respiratory cycle, without no–flow phases during the transition between inspiration and expiration. As a result, airway pressure declines gradually and nearly linearly throughout expiration, avoiding the abrupt pressure decay characteristic of conventional ventilation (5, 32).

By slowing expiratory pressure decline, FCV may stabilize end–expiratory lung volume, reduce alveolar collapse, and promote more homogeneous ventilation. These effects may be particularly advantageous during OLV, where heterogeneity of regional mechanics is amplified by surgical positioning, altered perfusion, and reduction of functional lung size (11, 25). By improving ventilation homogeneity, reducing cyclic derecruitment, and minimizing dissipated energy, active expiratory control may influence several mechanisms implicated in VILI.

While these theoretical considerations provide a compelling physiological rationale, implementation of FCV requires dedicated technology and a fundamentally different approach to ventilator management. Understanding these technical and practical aspects is essential before interpreting the growing body of preclinical and clinical evidence supporting FCV (33, 34).

## FCV: principles, technology, and ventilator management

### Fundamental principles of FCV

FCV applies constant flow during both inspiration and expiration, resulting in controlled, near–linear changes in intratracheal pressure throughout the respiratory cycle (9, 32, 35, 36). In contrast, expiration remains passive during VCV and PCV, with an initially rapid decline in expiratory flow and airway pressure.

### Intratracheal pressure monitoring

An additional distinguishing feature of FCV is the direct measurement of intratracheal pressure. Conventional ventilators estimate respiratory mechanics using airway pressures measured at the ventilator or proximal airway opening. However, particularly when narrow–diameter airways are used, substantial pressure losses may occur between the ventilator and the distal airway because of flow–dependent resistance.

FCV addresses this limitation through continuous intratracheal pressure monitoring. Pressure is measured via a dedicated lumen positioned close to the distal tip of the endotracheal tube, allowing real–time assessment of pressure immediately proximal to the carina. Consequently, the displayed pressures more accurately reflect the pressure experienced by the respiratory system than conventional proximal airway pressure measurements (25, 33).

This distinction is particularly relevant because FCV commonly employs small–bore airways associated with higher resistance. Without direct pressure measurement, substantial discrepancies between proximal and distal pressures could lead to inaccurate assessment of respiratory mechanics and inappropriate ventilator settings. Direct intratracheal pressure monitoring therefore represents a prerequisite for safe and accurate FCV.

### Minute ventilation in FCV

In conventional ventilation, tidal volume and respiratory rate are prescribed directly, whereas minute ventilation results from their product. In FCV, flow represents the primary variable used to control carbon dioxide elimination. Respiratory rate and tidal volume are not independently selected but emerge from the interaction between flow, PEEP, driving pressure, inspiratory to expiratory time ratio, and respiratory system compliance.

Consequently, clinicians accustomed to conventional ventilation frequently perceive the resulting respiratory pattern during FCV as unusual. Particularly during OLV, FCV often produces relatively low respiratory rates, larger tidal volumes, and lower overall minute ventilation than conventional ventilation while maintaining adequate carbon dioxide elimination. This phenomenon likely reflects improved ventilation efficiency and reduced dead–space ventilation, although the precise mechanisms remain incompletely understood (33).

### Compliance–guided ventilator titration

Because tidal volume depends directly on respiratory system compliance, FCV requires a fundamentally different approach to ventilator titration. Rather than targeting predefined tidal volume or pressure values, FCV is typically individualized according to dynamic respiratory system compliance (33, 37, 38).

Recent clinical guidance has proposed a stepwise compliance–guided strategy consisting of sequential optimization of PEEP and driving pressure (6). Following initiation of FCV, PEEP is adjusted in small increments while monitoring dynamic compliance and resulting tidal volume. The PEEP level associated with maximal compliance is subsequently selected as the individualized PEEP.

After identification of optimal PEEP, driving pressure is titrated in a similar fashion. Small stepwise adjustments are performed while monitoring changes in compliance and tidal volume generation. The driving pressure associated with maximal compliance and efficient tidal volume generation is then selected. Once pressure settings have been optimized, flow is adjusted to achieve the desired level of carbon dioxide elimination (33).

Thus, respiratory mechanics represent the primary titration target during FCV, while tidal volume and respiratory rate emerge from the interaction between pressure settings and compliance.

### Practical application during OLV

During thoracic surgery, FCV is commonly initiated using moderate PEEP and driving pressure values. Subsequent compliance–guided titration frequently results in pressure combinations that differ considerably between patients, reflecting individual variability in lung mechanics, positioning, and surgical conditions.

Clinical studies and reports have described that individualized FCV during OLV is associated with improved oxygenation, lower minute ventilation requirements, lower mechanical power, and more efficient carbon dioxide elimination compared with conventional ventilation strategies. Importantly, these physiological improvements are typically achieved without increasing airway pressures or compromising hemodynamic stability (11, 12).

The combination of direct intratracheal pressure monitoring, active expiratory control, and compliance–guided adjustment represents the core physiological concept underlying FCV. Whether these physiological advantages translate into meaningful clinical benefits has therefore become the focus of a growing body of experimental and clinical research (11, 38, 39).

### Enabling technologies: Evone ventilator and Tritube system

Clinical implementation of FCV requires dedicated technology capable of generating controlled inspiratory and expiratory flow while continuously monitoring intratracheal pressure. These requirements led to the development of the Evone® ventilator (Ventinova Medical, Eindhoven, The Netherlands) and the accompanying Tritube® airway system (34).

### The Evone ventilator

The Evone® ventilator was specifically designed to deliver FCV using the principles originally developed by Enk and colleagues (32). Active expiration is generated through the Venturi principle, enabling controlled negative expiratory flow (25, 32). In contrast to conventional ventilation, the transition between inspiration and expiration is associated with only minimal no–flow phases (9, 40).

A unique feature of the Evone® system is its integration of continuous intratracheal pressure monitoring. Rather than relying on airway pressures measured proximally within the ventilator circuit, pressure is measured close to the distal airway through a dedicated pressure lumen incorporated into the airway device. This allows accurate assessment of pressures experienced by the respiratory system and enables reliable calculation of dynamic respiratory system compliance during ventilation.

The ventilator interface differs from conventional anesthesia ventilators because flow, PEEP, and peak inspiratory pressure represent the primary variables selected by the clinician. Tidal volume and respiratory rate emerge dynamically from the interaction between these settings and respiratory system compliance. Consequently, operation of the Evone® system requires familiarity with the physiological principles underlying FCV and differs from traditional approaches to ventilator management (40, 41).

### The Tritube airway system

FCV is most commonly delivered through the Tritube®, a cuffed small–bore endotracheal tube with an outer diameter of approximately 4.4 mm. The Tritube® contains three separate lumens: a ventilation lumen, a cuff inflation lumen, and a dedicated pressure measurement lumen. The latter allows continuous monitoring of intratracheal pressure immediately proximal to the carina.

The small external diameter of the Tritube® was initially developed to facilitate upper airway and laryngotracheal surgery, where improved surgical exposure is frequently required. Compared with conventional endotracheal tubes, the Tritube® occupies substantially less airway space while maintaining cuff–based airway protection. Consequently, FCV was initially adopted primarily in airway surgery, where it demonstrated advantages over high–frequency jet ventilation by allowing continuous pressure monitoring, reliable carbon dioxide elimination, and protection against aspiration.

More recently, FCV has been increasingly investigated beyond airway surgery, including abdominal surgery, obesity, acute respiratory distress syndrome models, and thoracic surgery requiring OLV. In these settings, the Tritube® primarily serves as an enabling technology for precise pressure monitoring and delivery of FCV rather than as a means of improving surgical access (34, 42, 43).

### Advantages and limitations

However, important limitations should also be acknowledged. FCV currently requires dedicated equipment that is not widely available. Use of the Evone® system involves a learning curve for clinicians accustomed to conventional ventilation strategies. At present, administration of volatile anesthetics is not supported by the system, necessitating total intravenous anesthesia (TIVA). Furthermore, the Tritube® cannot currently accommodate flexible bronchoscopy through its ventilation lumen, which may limit its applicability in certain clinical scenarios. With the Conventional Tube Adapter (CTA)®, which enables delivery of FCV through conventional endotracheal tubes some of the limitations associated with the Tritube® can be overcome. Furthermore, FCV requires adequate neuromuscular blockade, as spontaneous respiratory efforts may interfere with active expiratory flow control and result in patient–ventilator dyssynchrony.

Despite these limitations, the Evone® platform remains the only commercially available ventilator capable of delivering true FCV in routine clinical practice. Consequently, it has served as the technological foundation for the growing body of experimental and clinical FCV research.

## From bench to bedside: preclinical evidence for FCV

The physiological rationale underlying FCV has been investigated extensively in experimental and translational studies over the past decade. These investigations have explored the effects of FCV on respiratory mechanics, gas exchange, lung aeration, energy dissipation, and histological markers of lung injury across a variety of experimental models. These studies provide the mechanistic foundation for subsequent clinical investigations (44).

### Mechanical energy dissipation as a mechanistic target

Barnes and Enk proposed that controlling both inspiration and expiration may reduce the amount of energy irreversibly transferred to lung tissue during each respiratory cycle. This concept is particularly relevant because dissipated energy has been implicated as a central determinant of VILI. Experimental work demonstrated that FCV generates lower hysteresis areas within pressure–volume loops compared with conventional ventilation, suggesting more efficient mechanical ventilation and lower energy dissipation.

These observations subsequently stimulated a series of experimental studies evaluating whether reduced dissipated energy translates into measurable improvements in pulmonary physiology and lung injury.

### FCV during OLV

The first dedicated investigations of FCV during OLV were performed in porcine models designed to replicate the physiological challenges encountered during thoracic surgery (9, 36, 45).

In a randomized crossover study, FCV was compared with pressure–regulated VCV during OLV. FCV resulted in significantly improved oxygenation, lower dead–space ventilation, and more efficient carbon dioxide elimination. Importantly, these physiological improvements were achieved despite lower minute ventilation requirements. Dynamic respiratory compliance was consistently higher during FCV, suggesting improved recruitment and more homogeneous ventilation distribution. Furthermore, FCV was associated with lower mechanical power, supporting the hypothesis that active expiratory control may reduce the mechanical burden imposed on the lung (36).

### Individualized FCV and respiratory mechanics

Beyond active expiratory control itself, several experimental studies have evaluated individualized compliance–guided FCV strategies.

In a prospective randomized porcine study, individualized FCV was compared with best clinical practice PCV. Ventilator settings during FCV were adjusted according to dynamic respiratory system compliance, whereas conventional ventilation followed contemporary protective ventilation principles. FCV resulted in superior oxygenation, lower respiratory system elastance, and improved ventilation efficiency. Most importantly, these physiological benefits were observed despite lower minute ventilation requirements and reduced airway pressures (9).

These findings supported the subsequent development of structured compliance–guided FCV titration protocols (6).

### Acute respiratory distress syndrome models

The potential lung–protective properties of FCV have also been investigated in experimental acute respiratory distress syndrome (ARDS).

Individualized FCV was evaluated in a porcine oleic acid–induced ARDS model and compared it with individualized pressure–controlled ventilation. FCV resulted in significantly improved oxygenation and respiratory system compliance while reducing mechanical power requirements. Computed tomography analyses demonstrated improved lung aeration and more homogeneous distribution of ventilation. Importantly, FCV achieved these benefits without increasing airway pressures or hemodynamic instability (8).

### Lung aeration and imaging studies

Several experimental investigations have used imaging techniques to evaluate the effects of FCV on regional lung aeration.

Studies employing computed tomography and electrical impedance tomography demonstrated more homogeneous ventilation distribution during FCV compared with conventional ventilation modes. Improved preservation of aerated lung volume and reductions in poorly aerated or non–aerated regions have been reported across multiple experimental settings. These findings support the hypothesis that active expiratory control may reduce cyclic derecruitment and improve maintenance of end–expiratory lung volume (9, 39, 40, 46).

More recently, investigations in models of increased intra–abdominal pressure demonstrated that FCV improved ventilation distribution and respiratory mechanics under conditions associated with substantial reductions in lung compliance.

### Histological evidence of lung protection

Although most experimental studies were not primarily designed to evaluate histological injury, several investigations have reported findings consistent with reduced lung injury during FCV.

Compared with conventional ventilation, FCV has been associated with reduced alveolar edema, less inflammatory cell infiltration, and preservation of normal alveolar architecture (44). These findings generally parallel improvements observed in oxygenation, compliance, and aeration. However, histological endpoints have varied substantially across studies, limiting direct comparison between experimental models (47).

Importantly, no experimental study has yet definitively demonstrated that FCV prevents VILI. Rather, the available evidence suggests that FCV favorably influences several physiological mechanisms implicated in lung injury, including energy dissipation, ventilation heterogeneity, alveolar instability, and mechanical power.

### Summary of preclinical evidence

Taken together, preclinical studies have reported favorable effects of FCV on gas exchange, respiratory mechanics, mechanical power, energy dissipation, and lung aeration across different experimental models. However, these findings are predominantly based on short-term physiological endpoints, and their translation into clinically meaningful benefits remains uncertain (9, 35, 36).

## Clinical evidence for FCV in thoracic anesthesia and OLV

The translation of FCV from experimental models into clinical practice has occurred gradually over the past decade. Initial investigations focused primarily on airway surgery, where the unique characteristics of the Tritube® system offered practical advantages compared with conventional airway management techniques (34). More recently, clinical research has expanded into thoracic anesthesia and OLV, where the physiological benefits observed in preclinical studies can be evaluated in a setting characterized by substantial respiratory challenges and high risk of pulmonary complications (Table 1).

**Table 1:** Study–level information on studies of FCV in thoracic anesthesia with OLV.

Study	Design/population	*n*	Comparator and key settings	Main quantitative findings
Schranc et al. (36)	Randomized experimental crossover study; porcine OLV	10; FCV *n* = 5, PRVC *n* = 5	PRVC	During OLV, FCV increased PaO_2_ from 153.7 ± 12.7 to 169.9 ± 15.0 mmHg (*P* = 0.002) and reduced PaCO_2_ from 53.0 ± 11.0 to 43.2 ± 6.0 mmHg (*P* < 0.001). Respiratory elastance was lower with FCV (77.2 ± 10.5 vs. 103.7 ± 9.5 cmH_2_O/L, *P* < 0.001) and peak inspiratory pressure was lower (22.0 ± 2.3 vs. 27.4 ± 1.9 cmH_2_O, *P* < 0.001). No significant difference in lung aeration or haemodynamics.
Abram et al. (11)	Single–centre randomized controlled trial; patients undergoing thoracic surgery with OLV	43; FCV *n* = 21, PCV *n* = 22	Individualized FCV vs. conventional PCV; FCV pressure settings individualized according to respiratory system compliance	PaO_2_/FiO_2_ after 30 min OLV was higher with FCV: 187 vs. 136 mmHg, median difference 39 mmHg (95% CI 1–75), *P* = 0.047. FCV also required lower minute ventilation for CO_2_ elimination and was associated with lower mechanical power.
Wittenstein et al. (48)	Two–centre randomized controlled clinical trial; thoracic surgery requiring >30 min OLV	78; FCV *n* = 39, VCV *n* = 39	FCV vs. VCV; both groups: VT 5 mL/kg PBW, PEEP 5 cmH_2_O, FiO_2_ 1.0, RR adjusted to normocapnia	PaO_2_ after 20 min OLV did not differ: 24.8 ± 14.8 vs. 26.1 ± 15.9 kPa, mean difference −1.2 kPa (95% CI −8.2 to 5.7), *P* = 0.721. Mechanical power was lower with FCV at 20 min OLV (6.0 ± 1.5 vs. 7.4 ± 2.5 J/min, *P* = 0.003). No relevant differences in gas exchange, rescue manoeuvres, or postoperative outcomes.
Hanselmann et al. (12)	Case series; patients undergoing thoracic surgery with OLV	3	FCV used during OLV; comparison with preceding/conventional PCV within the clinical course	FCV maintained adequate ventilation during OLV without repeated recruitment manoeuvres and was associated with improved gas exchange and stable haemodynamics. As a three–patient case series, no comparative group–level effect estimates were reported.

FCV, flow–controlled ventilation; OLV, one–lung ventilation; PCV, pressure–controlled ventilation; PBW, predicted bodyweight; PEEP, positive end–expiratory pressure; PRVC, pressure–regulated volume–controlled ventilation; RR, respiratory rate; VT, tidal volume.

### Early clinical experience

The earliest clinical applications of FCV occurred primarily in upper airway and laryngotracheal surgery (49). In these procedures, the small external diameter of the Tritube® provided improved surgical exposure while maintaining a sealed airway and enabling continuous intratracheal pressure monitoring.

Several observational studies reported effective oxygenation and carbon dioxide elimination during complex airway procedures, while avoiding some limitations associated with high–frequency jet ventilation (HFJV), including impaired carbon dioxide clearance, inability to continuously monitor airway pressure, and risk of aspiration. These early investigations established the feasibility and safety of FCV in clinical practice and provided important experience with the Evone® platform (42, 43).

Although these studies were not performed during OLV, they demonstrated that active control of expiration could be implemented safely in humans and encouraged exploration of FCV in other clinical settings.

### Early thoracic applications

Initial clinical evidence consisted primarily of case reports and small case series. These reports described successful implementation of FCV during thoracic procedures requiring OLV and described improvements in oxygenation and ventilation efficiency. In several cases, FCV enabled maintenance of adequate oxygenation at substantially lower inspired oxygen fractions than would typically be required during conventional ventilation. Furthermore, satisfactory carbon dioxide elimination was achieved despite comparatively low minute ventilation and respiratory rates (12).

While inherently limited by their observational nature, these reports provided important proof–of–concept evidence and demonstrated the feasibility of FCV in routine thoracic anesthesia practice.

### The first randomized clinical trials during OLV

In one of the first randomized controlled trials evaluating FCV during thoracic surgery in humans, patients undergoing thoracic surgery requiring OLV were randomized to receive either FCV or PCV (11).

FCV resulted in significantly improved oxygenation throughout OLV, reflected by higher arterial oxygen tensions and superior oxygenation indices. At the same time, carbon dioxide elimination was more efficient despite lower minute ventilation requirements. Respiratory system compliance was higher during FCV, while mechanical power was significantly reduced compared with conventional ventilation.

Perhaps most importantly, these physiological improvements were achieved without evidence of hemodynamic compromise or increased adverse events.

Another randomized controlled trial, explored these effects by comparing FCV with VCV during OLV. In contrast to the earlier trial, the PROFLONE investigators standardized tidal volumes (5 mL/kg) and PEEP (5 cm H_2_O) across both groups, thereby isolating the impact of the ventilatory flow profile itself. Under these matched conditions, oxygenation did not differ significantly between FCV and VCV. Nevertheless, FCV was associated with a significant reduction in mechanical power compared with VCV, indicating improved respiratory mechanics independent of oxygenation changes (48). These results suggest that the mechanical power advantage of FCV is an intrinsic property of its constant inspiratory and expiratory flow, while the oxygenation benefit observed in the earlier trial may have been influenced, at least in part, by differences in tidal volume between groups.

### Oxygenation and ventilation efficiency

Across clinical studies, FCV has generally been associated with preserved or improved gas exchange despite comparatively low minute ventilation requirements. However, oxygenation findings have not been uniform, as demonstrated by the PROFLONE trial, in which oxygenation did not differ between FCV and VCV under matched tidal volume and PEEP conditions (11, 35, 36).

### Safety and feasibility

Across published studies, FCV has demonstrated a favorable safety profile.

No consistent increase in hemodynamic instability, severe hypercapnia, hypoxemia, or device–related complications have been reported (12, 50).

From a practical perspective, implementation of FCV requires familiarity with the unique ventilation philosophy of the Evone® platform and compliance–guided titration strategies. However, recent findings have demonstrated the feasibility of integrating FCV into anesthesia workflows (11, 38, 39).

### Comparison with HFJV and other advanced modes

Although FCV was initially investigated in airway surgery, where HFJV represents an established alternative, HFJV is not routinely used for OLV and direct comparison is therefore of limited relevance to thoracic anesthesia. Whether FCV offers advantages over other contemporary OLV strategies, including individualized driving pressure–guided or open–lung approaches, remains unknown (43, 51–53).

## Limitations, knowledge gaps, and future directions

Perhaps the most important limitation of the current FCV literature is the predominance of physiological endpoints. Improvements in oxygenation, respiratory compliance, carbon dioxide elimination, ventilation homogeneity, and mechanical power have been reported across experimental and clinical studies. However, these surrogate outcomes do not necessarily predict reductions in PPCs or improvements in longer–term clinical outcomes.

This distinction is particularly relevant in thoracic anesthesia. Several contemporary ventilation trials have demonstrated substantial improvements in intraoperative physiology without corresponding reductions in PPCs. Examples include studies evaluating individualized PEEP, driving pressure–guided ventilation, and open–lung approaches (16, 54–56). Consequently, although FCV appears physiologically attractive, its true clinical value cannot be inferred solely from improvements in respiratory mechanics or gas exchange (16, 54, 56).

Furthermore, most clinical investigations evaluating FCV remain relatively small. The majority were conducted at single centers and enrolled highly selected patient populations. As a result, external validity remains limited, and the reproducibility of findings across different institutions, surgical procedures, and patient populations requires further evaluation.

Another important limitation is the heterogeneity of comparator ventilation strategies. Studies have compared FCV with VCV, PCV, pressure–regulated VCV, and individualized protective ventilation approaches. Differences in tidal volume, PEEP strategies, recruitment maneuvers, and oxygen administration complicate direct comparison between studies and may contribute to variability in observed treatment effects (11, 36, 38, 39).

### Standardization of FCV protocols

Although FCV is often discussed as a distinct ventilation mode, substantial variation currently exists regarding its clinical implementation.

Published studies differ in their approaches to flow selection, PEEP titration, driving pressure optimization, and carbon dioxide management. While recent compliance–guided titration strategies provide a more structured framework for clinical application, universal standards have not yet been established (6). Consequently, it remains unclear which components of the FCV concept contribute most strongly to the observed physiological benefits.

An additional challenge relates to the distinction between the ventilatory mode itself and the individualized ventilation strategy commonly used alongside FCV. Many studies compare individualized FCV with conventional ventilation using fixed ventilator settings. Therefore, some observed benefits may result from improved individualization rather than active expiratory control alone. Future investigations should seek to disentangle these effects and determine the relative contribution of expiratory flow control, intratracheal pressure monitoring, and compliance–guided titration.

### Mechanical power and energy dissipation: emerging targets

Increasing evidence suggests that cumulative energy exposure may play an important role in the development of VILI. FCV may reduce mechanical power requirements in both experimental and clinical studies, largely through improved ventilation efficiency and lower minute ventilation requirements. Whether this reduction in mechanical power translates into lower biological injury remains unknown (31, 32).

Furthermore, conventional mechanical power calculations primarily focus on inspiratory energy delivery and incompletely account for expiratory mechanics. Because FCV was specifically designed to modify expiratory flow dynamics and reduce dissipated energy, future research may require more sophisticated metrics capable of quantifying the contribution of expiration to lung injury. Such investigations could improve understanding of the mechanistic relationship between FCV, energy transfer, and VILI (3).

### Integration into personalized ventilation strategies

The evolution of perioperative ventilation increasingly favors individualized physiological approaches rather than universal ventilator settings. In many respects, FCV aligns naturally with this paradigm.

The ability to continuously monitor intratracheal pressure and dynamically assess respiratory system compliance creates opportunities for highly individualized ventilator management. Future developments may further integrate FCV with bedside physiological monitoring tools, including electrical impedance tomography, lung ultrasound, automated compliance analysis, and advanced respiratory mechanics assessment.

Rather than replacing established protective ventilation principles, FCV may ultimately become one component of broader personalized ventilation strategies that integrate recruitment assessment, oxygen management, mechanical power reduction, and postoperative respiratory support.

### Practical implementation and health–economic considerations

Several practical barriers currently limit widespread implementation of FCV, including the need for dedicated ventilators and proprietary disposables, as well as specific training in a ventilation approach that differs substantially from conventional modes.

The Evone® platform remains dependent on total intravenous anesthesia because inhalational anesthetic delivery is not supported. In addition, the current system does not provide conventional spontaneous breathing modes, limiting its applicability in clinical scenarios where assisted ventilation, spontaneous ventilation, or weaning strategies are desirable. These restrictions are particularly relevant in intensive care medicine, where transition between controlled and assisted ventilation represents an integral component of routine respiratory support. Consequently, future development of FCV technology will need to address not only physiological performance but also practical flexibility and integration into established perioperative and critical care workflows. Fourth, the application of FCV during thoracic surgery also presents unique airway management challenges. While FCV was originally developed for use with the Tritube®, thoracic procedures requiring OLV generally necessitate lung isolation using either double–lumen tubes or bronchial blockers. As a result, current clinical implementation of FCV during OLV requires a dedicated airway adapter, which is compatible with double–lumen tubes. Furthermore, the small internal diameter of the Tritube® precludes routine passage of a flexible bronchoscope, which may complicate airway inspection and management in selected situations. Although recent studies have demonstrated the feasibility of FCV during OLV, wider adoption will likely depend on further technological developments that facilitate integration with established thoracic airway management techniques while preserving the physiological advantages of active expiratory control.

Economic considerations remain largely unexplored. While FCV may improve intraoperative physiology, its effects on hospital length of stay, intensive care utilization, resource consumption, and overall healthcare costs remain unknown. Future multicenter trials should therefore incorporate health–economic analyses alongside clinical outcome measures.

### Future clinical trials

The most important priority for future research is the evaluation of FCV in adequately powered randomized clinical trials using clinically relevant outcome measures.

Future studies should move beyond short–term physiological endpoints and investigate whether FCV influences PPCs, respiratory failure, intensive care admission, hospital length of stay, quality of recovery, and mortality. Given the relatively low incidence of these outcomes, multicenter collaborative studies will likely be required.

Such trials should also evaluate FCV within contemporary perioperative care pathways rather than as an isolated intraoperative intervention. Increasing evidence suggests that pulmonary outcomes are influenced by the cumulative interaction of intraoperative ventilation, postoperative respiratory support, analgesia, mobilization, and fluid management. Consequently, the role of FCV may be best understood within integrated perioperative respiratory care bundles.

### Future technological developments

The concept underlying FCV may also serve as a foundation for future ventilatory innovations. Advances in computational physiology, machine learning, and automated ventilator control could enable continuous adjustment of flow, PEEP, and driving pressure according to real–time respiratory mechanics. Such systems may facilitate highly individualized ventilation strategies that adapt dynamically to changing physiological conditions during surgery.

## Practical implementation and health–economic considerations

In addition to these technological limitations, implementation of FCV requires specific training and adaptation of established clinical workflows. Ventilator management differs substantially from conventional ventilation, as flow rather than respiratory rate represents the principal variable for controlling minute ventilation, while PEEP and driving pressure are individualized according to respiratory system compliance. During OLV, integration into established airway management workflows represents an additional challenge, particularly when lung isolation, bronchoscopic confirmation of tube or blocker position, or intraoperative airway interventions are required. These practical considerations may currently limit broader implementation of FCV, particularly in centers without previous experience with the technique.

## Discussion

Across experimental and early clinical studies, FCV has shown relatively consistent favorable effects on respiratory mechanics, gas exchange, and mechanical power. However, the clinical evidence remains limited and predominantly based on physiological endpoints (11, 35, 36, 48). A recurring observation is that adequate carbon dioxide elimination can be achieved during FCV despite comparatively low minute ventilation requirements, suggesting improved ventilatory efficiency (32). However, the central question remains unanswered: do these physiological advantages translate into improved clinical outcomes?

This question is particularly important because recent thoracic ventilation research has repeatedly demonstrated a disconnect between improvements in intraoperative physiology and postoperative outcomes. Individualized PEEP strategies, recruitment maneuvers, open–lung approaches, and driving pressure–guided ventilation have all improved respiratory mechanics and oxygenation under certain conditions. Yet, large randomized trials have generally failed to demonstrate consistent reductions in PPCs. These experiences serve as an important reminder that physiological plausibility alone is insufficient to establish clinical benefit (3, 31, 54).

Several explanations may account for this apparent discrepancy. First, pulmonary complications following thoracic surgery are multifactorial and extend beyond intraoperative ventilation alone. Postoperative pain, impaired cough, fluid administration, surgical trauma, mobilization, pre–existing lung disease, and postoperative respiratory support all contribute substantially to pulmonary outcomes (2). Consequently, even meaningful improvements in intraoperative respiratory mechanics may exert only modest effects on overall PPC risk.

Second, current physiological metrics may incompletely capture the mechanisms responsible for lung injury. Variables such as oxygenation, compliance, and mechanical power provide valuable insight into respiratory physiology but may not fully reflect the complex biological processes leading to PPCs (4, 57–59). It remains possible that FCV influences pathways that are not adequately measured by conventional physiological endpoints, just as it remains possible that improvements in these endpoints are insufficient to alter clinical outcomes.

Third, the current clinical evidence remains underpowered to detect differences in patient–centered outcomes. Most FCV studies enrolled relatively small patient populations and were designed primarily to evaluate feasibility and physiological efficacy. Consequently, the absence of outcome data should not be interpreted as evidence of absent benefit but rather as evidence of insufficient investigation (11, 38, 39).

The relationship between FCV and mechanical power deserves particular attention. Mechanical power has emerged as one of the most influential concepts in contemporary respiratory physiology because it integrates multiple ventilatory variables into a single estimate of energy transferred to the respiratory system. Several FCV studies have reported reductions in mechanical power compared with conventional ventilation strategies. However, FCV may also highlight limitations of current mechanical power concepts. Existing calculations largely focus on inspiratory energy delivery and provide limited information regarding expiratory mechanics or dissipated energy (30, 60). From a broader perspective, FCV may ultimately be most important not because of the specific technology itself, but because of the physiological questions it raises. The development of FCV has renewed interest in expiratory mechanics, alveolar stability, ventilation homogeneity, and energy dissipation–topics that historically received considerably less attention than inspiratory variables (32, 61).

Whether FCV will ultimately become part of routine thoracic anesthesia practice remains uncertain. The technology currently requires dedicated equipment, specialized disposables, and familiarity with a distinct ventilation philosophy. These barriers may limit widespread adoption unless future studies demonstrate clinically meaningful benefits. Nevertheless, even if FCV itself does not achieve broad implementation, the physiological principles underlying active expiratory control may influence future ventilator development and inspire new approaches to individualized respiratory support.

## Conclusion

Taken together, the current evidence suggests that FCV should not be viewed simply as another ventilatory mode competing with VCV or PCV. Rather, it represents a different physiological paradigm that redefines expiration as a potentially modifiable determinant of lung protection. The consistency of physiological benefits observed across experimental and clinical studies warrants further investigation and provides a strong rationale for adequately powered outcome–oriented trials in thoracic anesthesia.
